# Analysis of Papaya Cell Wall-Related Genes during Fruit Ripening Indicates a Central Role of Polygalacturonases during Pulp Softening

**DOI:** 10.1371/journal.pone.0105685

**Published:** 2014-08-27

**Authors:** João Paulo Fabi, Sabrina Garcia Broetto, Sarah Lígia Garcia Leme da Silva, Silin Zhong, Franco Maria Lajolo, João Roberto Oliveira do Nascimento

**Affiliations:** 1 Department of Food Science and Experimental Nutrition, FCF, University of São Paulo, São Paulo, São Paulo, Brazil; 2 State Key Laboratory of Agrobiotechnology, School of Life Sciences, The Chinese University of Hong Kong, Hong Kong, China; 3 University of São Paulo, – NAPAN – Food and Nutrition Research Center, São Paulo, São Paulo, Brazil; University of Malaga-Consejo Superior de Investigaciones Científicas, Spain

## Abstract

Papaya (*Carica papaya* L.) is a climacteric fleshy fruit that undergoes dramatic changes during ripening, most noticeably a severe pulp softening. However, little is known regarding the genetics of the cell wall metabolism in papayas. The present work describes the identification and characterization of genes related to pulp softening. We used gene expression profiling to analyze the correlations and co-expression networks of cell wall-related genes, and the results suggest that papaya pulp softening is accomplished by the interactions of multiple glycoside hydrolases. The polygalacturonase *cpPG1* appeared to play a central role in the network and was further studied. The transient expression of *cpPG1* in papaya results in pulp softening and leaf necrosis in the absence of ethylene action and confirms its role in papaya fruit ripening.

## Introduction

Papaya (*Carica papaya* L.) is a fleshy fruit that undergoes severe pulp softening during ripening. Although softening increases the sensory and nutritional properties of the fruit, it also contributes to post-harvest deterioration and product losses because the fruit becomes susceptible to physical injury and mold growth [1].

The softening of fleshy fruits during ripening is characterized by the degradation of the plant's cell wall, which is mainly formed by polysaccharides organized in the pectic (amorphous) and the hemicellulosic/cellulosic (crystalline) fractions and the loss of the rigidity of cell wall structure is attributed to the solubilization of both fractions [2]. This solubilization is achieved by the interactive action of several enzymes on determined polysaccharides, such as polygalacturonases (PGs), pectinesterases (PMEs) and pectate lyases (PLs) on homogalacturonans, arabinofuranosidases (ARFs) and galactanases (GALs) on heterogalacturonans and xyloglucan endotransglucosylases/hydrolases (XTHs), endoxylanases (EXYs) and cellulases (CELLs) on hemicelluloses [3], [4].

In the case of papaya fruit, the investigation of polysaccharide changes during ripening has revealed that the disassembly of the cell wall is predominately caused by the degradation and solubilization of pectin [5]. In this regard, polygalacturonase (*cpPG1*) [6] and β-galactosidase (*cp_b-GAL*) [7] play a central role in pectin solubilization during papaya ripening. However, other studies have provided evidence that the solubilization of the hemicellulosic fraction by endoxylanases (EXY) is a major contributor to the softening of papaya fruit [8]. This apparent contradiction between the experimental evidence provided by different studies suggests that the disassembly of the cell wall in papaya fruit is a more complex process that involves several enzymes and various cell wall components. In fact, the investigation of several genes or proteins simultaneously may provide better insight on the physiological process, as revealed by data from an XSpecies microarray of papaya ripening that indicated that several cell wall-related genes exhibit similarities to the gene expression profiles observed during the transition of 5- to 11-day-old hypocotyls in *Arabidopsis thaliana* [9], [10]. Moreover, evaluating the correlations and co-expression of a set of genes may provide clues to the interactive role of those genes.

Therefore, investigating the network of cell wall-related genes, specifically those encoding depolymerization or degradation enzymes, may further elucidate the mechanism of cell wall disassembly during papaya ripening. Thus, this study describes the qPCR analysis of the mRNA levels of some genes of enzymes that act on homogalacturonans (polygalacturonases, pectinesterases and pectate lyases), heterogalacturonans (arabinofuranosidases and galactanases) and hemicellulose (xyloglucan endotransglucosylases/hydrolases and xylanases), which covers the diversity of polysaccharides from the amorphous and crystalline fractions of cell wall, and the correlations and co-expression network of 25 cell wall-related genes during papaya ripening. In addition, the protein levels and enzymatic activity of endopolygalacturonase and the overexpression of the c*pPG1* gene were measured in agroinfiltrated papaya fruit pulp and leaves.

## Results

### Cloning cell wall-related genes in papaya fruit

**Figure 1:**
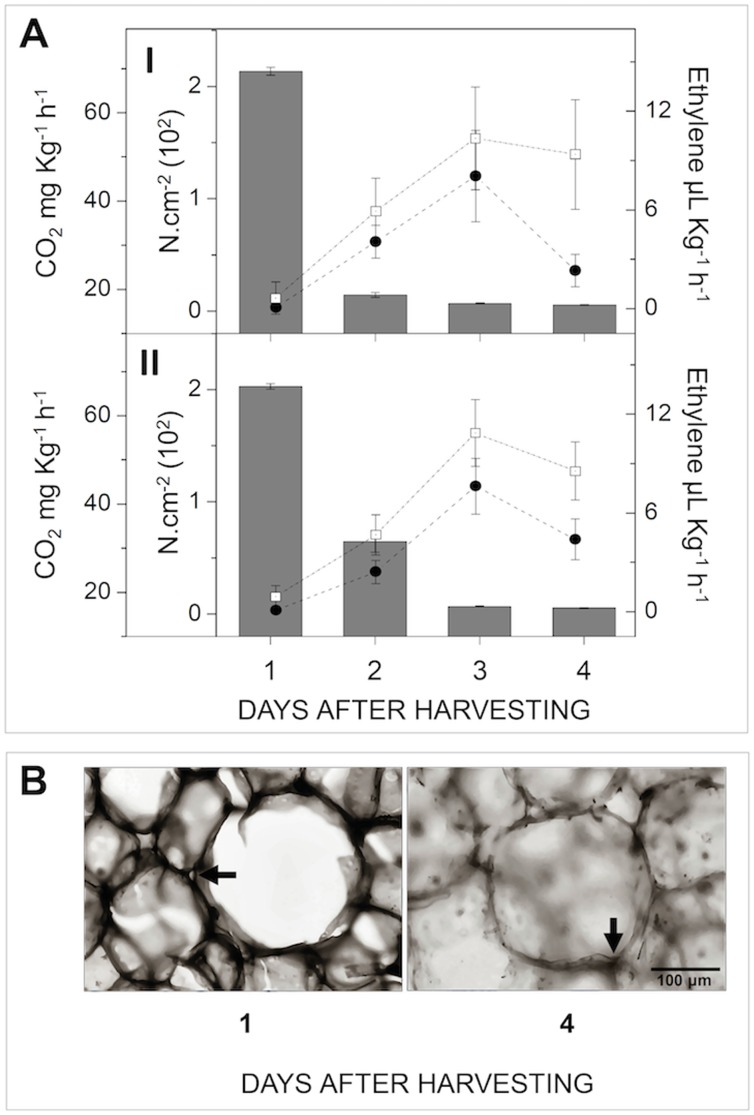
Ripening of papaya fruit. **A**) The amount of CO_2_ produced by respiration (Open squares – mg Kg^−1^ h^−1^), the production of endogenous ethylene (Black circles - µL Kg^−1^ h^−1^), and the pulp firmness (Bars – N.cm^−2^) were monitored throughout ripening. Error bars indicate SDs of the mean (*n* = 12) for each sampling (**I** and **II**). **B**) Sections of frozen papaya pulp (12–15 µm) in two different stages (unripe and ripe – 1^st^ and 4^th^ day after harvesting, respectively) were stained with 0.05% of toluidine blue and documented using light microscope. Different colorations indicate cell wall thinning. Arrows indicate the junction zones of cell walls. Bar indicates the scale. The image is a representation of, at least, a triplicate of experiment.

The fruit used for the cloning and expression analysis presented typical softening resulting from the extensive solubilization of cell wall components, as indicated by the climacteric decrease in pulp firmness [1] and cell wall thinning (**Figure 1**).

**Figure 2:**
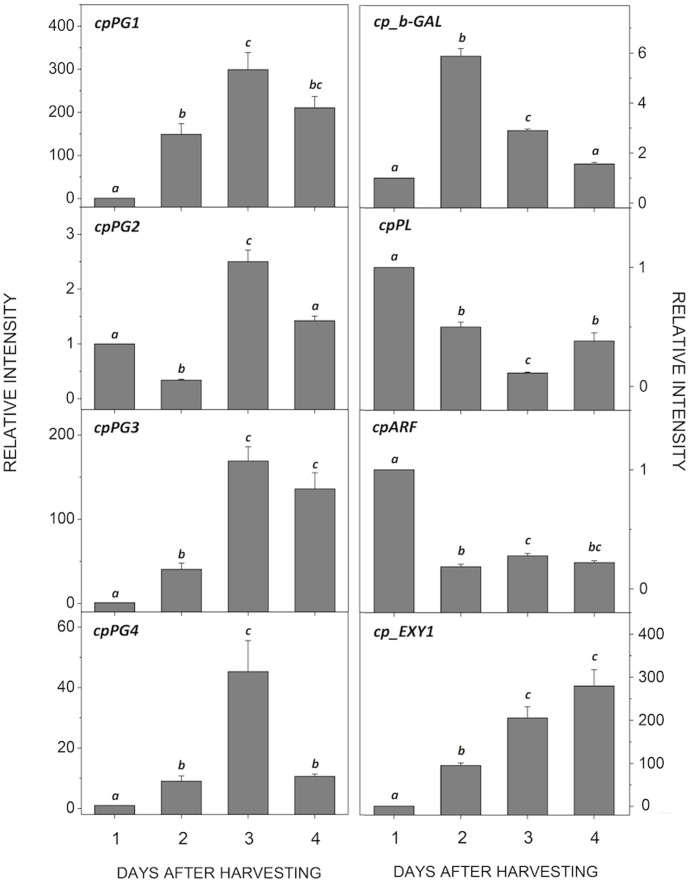
Expression of cell wall-related genes during papaya ripening. Real-time PCR (qPCR) was used to analyze the mRNA levels of various genes during ripening. The column heights indicate the relative mRNA abundance; the expression values for unripened fruit one day after harvest were set to 1. The error bars on each column indicate the SD of four technical replicates from samplings I and II. The different letters represent samples that were significantly different from those collected on other days post-harvest (within the same gene), as determined by one-way ANOVA and Tukey's test (α<0.05, *n* = 4).

A BLAST search of the papaya genome allowed the cloning of *cpPG1* (**FJ007644** – Fabi et al., 2009), *cpPG2* (**GQ479794**), *cpPG3* (**GQ479795**), *cpPG4* (**GQ479796**), *cpARF* (**GQ479793**), the previously reported *cp_b-GAL* (**AF064786** – [7]) and *cpPL* (**DQ660903**). As summarized in **Table 1**, the properties of the encoded proteins are similar to those from other plants. The four PGs were differentially expressed and peaked in the climacteric papaya (3DAH) (**Figure 2**). *CpEXY1* was also differentially expressed during ripening, but in a linear increasing manner. Papaya *cp_b-GAL* was also up-regulated, although it was brought to the initial levels after second day after harvesting (2DAH), whereas both *cpPL* and *cpARF* were down-regulated. When the amount of the transcripts of the up-regulated genes was compared, *cpPG1* and *cp_b-GAL* were determined to be the most abundant (**Figure S1 in File S1**), and the amount of *cpPG1* exceeded that of the less abundant *cpPG4* by six orders of magnitude.

**Table 1:** Cell wall-related proteins identified in papaya pulp and their probable biological properties.

*Gene name*	*Length* †	*Accession Number*	*Chromosomes*	*TAIR*	*Cleavage Site*	*Molecular Weight (KDa)^†^*	*Isoelectric Point* ††	*Blast Best Match*
cpPG1	397	**ACH82233**	Un contig 23855 (ABIM01023821)	AT3G59850	Ala^29^	38.98	9.14	Polygalacturonase [P. communis] CAH18935
cpPG2	494	**ACV85695**	LG4 contig 16510 (ABIM01016487)	AT3G57510	Ser^33^	51.03	5.21	Polygalacturonase A [A. chinensis] AAF71160
cpPG3	444	**ACV85696**	LG4 contig 19176 (ABIM01019150)	AT3G07970	Gly^31^	45.78	9.22	Polygalacturonase QRT2 [A. thaliana] NP_187454
cpPG4	389	**ACV85697**	LG1 contig 4651 (ABIM01004644)	AT2G43870	Ala^31^	38.67	8.54	Polygalacturonase 3 [P. communis] BAF42034
cpARF	710	**ACV85694**	LG4 contig 9013 (ABIM01009003)	AT3G10740	Ala^43^	74.16	4.94	α-L-arabinofuranosidase [M. domestica] AAP97437
cp-b-GAL	721	**AAC77377**	LG7 contig 16621 (ABIM01016598)	AT3G13750	Ala^21^	78.49	8.26	β-galactosidase 1 [P. persica] ABV32546
cpPL	369	**ABG66730**	LG6 contig 2998 (ABIM01002995)	AT3G07010	Ser^19^	40.52	9.14	Pectate lyase [P. persica] BAF43573

† Amino acids numbers;

†† The Molecular Weight and isoelectric points were estimated using the mature proteins.

### Gene expression and correlation analysis

**Figure 3:**
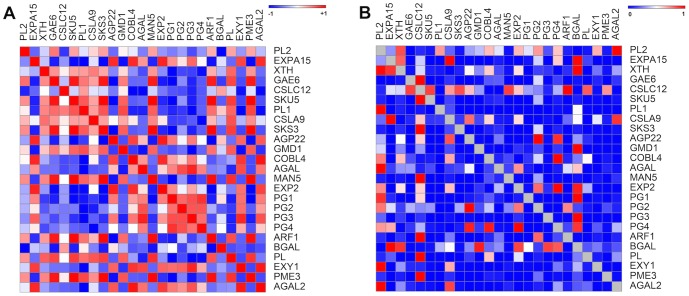
Pearson correlations and associated *p*-values of papaya cell wall-related genes. The R package for weighted correlation network analysis (weighted gene co-expression network analysis - WGCNA) was used to calculate the Pearson correlation (**Figure A**) and the corresponding *p*-values for the 25 papaya cell wall-related genes that were identified by our group (**Figure B**). From the 600 possible gene expression correlations, 300 had *p*-values less than or equal to 0.05 and were further analyzed. **Figure 1** **A**: The heat map is described as positive values set to red color and negative values set to blue color. **Figure** **B**: The heat map is described as values near to one set to red color and values near to zero set to blue color. The twenty-five genes are described in **Table S1 in File S1**.

**Figure 4:**
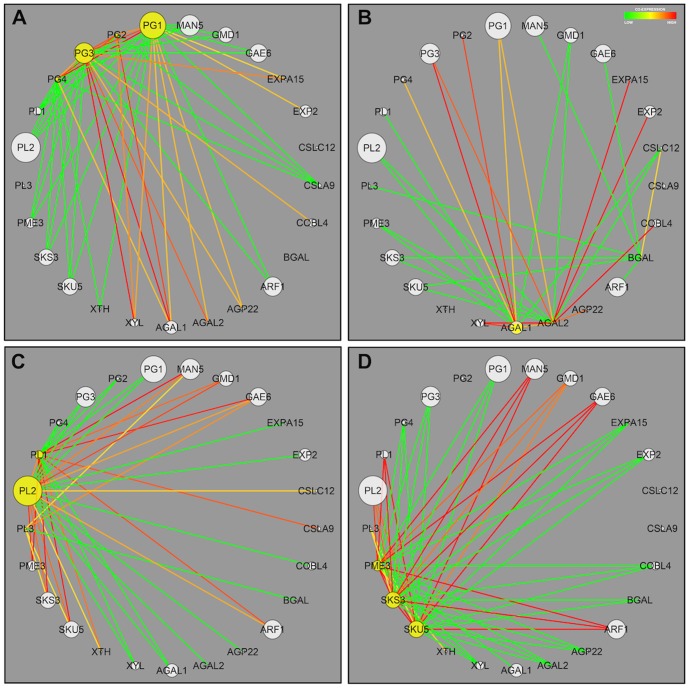
Co-expression network of papaya cell wall-related genes given by the weighted gene co-expression network analysis (WGCNA). The R package of the weighted gene co-expression network analysis (WGCNA) program was used to produce a gene association network for 25 papaya cell wall-related genes. The size of the circle indicates the weight of the gene on the network. In **Figures A, B, C** and **D**, the genes considered for the network assembly are highlighted with yellow backgrounds. **Figure A** describes the network for the papaya *PG* genes. **Figure B** describes the network for the papaya *GAL* genes. **Figure C** describes the network for the papaya *PME* genes. **Figure D** describes the network for the papaya *PL* genes. The color lines are described as positive co-expression values set to red color and negative co-expression values set to green color. The twenty-five genes are described in **Table S1 in File S1**.

The expression pattern of a collection of 25 cell wall-related genes that were previously described as ripening-related ones [11], [12], [10] was analyzed for co-expression during ripening. Among the 600 possible correlations, 300 had *p*-values less than 5% and were further analyzed (**Figure 3**), which revealed that PGs, galactanases, PMEs and PLs significantly impact the network assembly (**Figure 4**). The PGs were positively correlated to galactanases, xylanases and, to a lesser degree, expansins and negatively correlated to a second group of genes encompassing PMEs, PLs and ARF. Because the expression of papaya cell wall disassembling genes was similar to *Arabidopsis* from 5 to 11-day-old hypocotyls growth [9], [10], a new comparison of papaya data was done with newly *Arabidopsis* data [13]. The comparison (**Table S1 in File S1**) showed changes in the expression of 11 out of 16 genes, which were similar between the ripening fruit and the growing plant. The genes that were most significantly affected in both developmental processes were *cpPG1* and its homologues.

### Transient expression of papaya *cpPG1*

**Figure 5:**
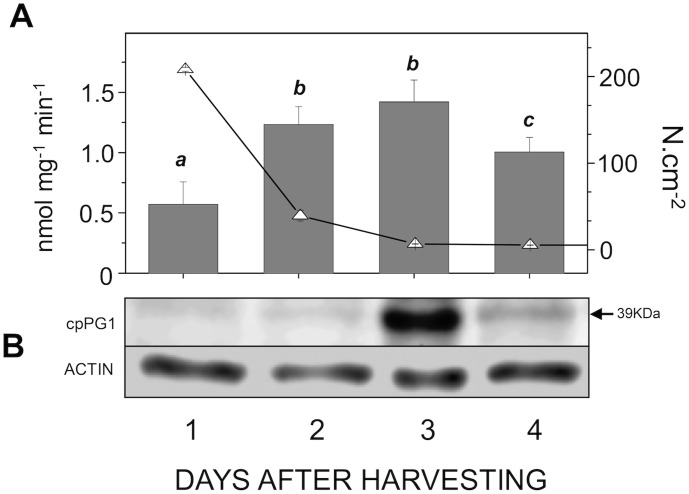
Correlation between pulp softening, enzymatic activity and cpPG1 protein expression during papaya ripening. **A**) The pulp firmness (open triangles) was monitored during the ripening process using a texturometer (Fabi et al., 2007), and the measurements are given by N.cm^−2^. PG enzymatic activities was done according to Fabi et al. (2009). The error bars indicate the SDs of the mean. The different letters represent samples that were significantly different from those collected on other days post-harvest, as determined by one-way ANOVA and Tukey's test (α<0.05, *n* = 4). **B**) Western blotting experiment was done using the monoclonal anti-actin for plants as a control experiment (ACTIN), and the images are representative of at least a triplicate experiment. The arrow indicates the molecular weight of the mature protein cpPG1 with the corresponding molecular weight.

We identified four papaya PG genes that have AT-rich intronic regions with two specific patterns, one for *cpPG1* and *cpPG4* and another for *cpPG2* and *cpPG3* (**Figure S2 in File S1**). Notably, the proteins encoded by *cpPG1*/*cpPG4* were separated in a distinct clade from *cpPG2*/*cpPG3* when the PGs from diverse plants were aligned (**Figure S3 in File S1**). The expression of *cpPG1* in prokaryotic cells resulted in a recombinant product with the expected size and identity of the predicted cpPG1 protein, as revealed by MS sequencing (**Figure S4 in File S1**). This protein was used to produce polyclonal antibodies utilized in western blotting (**Figure S5 in File S1**), which showed that the amount of PG protein was correlated to that of the *cpPG1* transcript and its enzymatic activity and with pulp softening (**Figure 5**).

**Figure 6:**
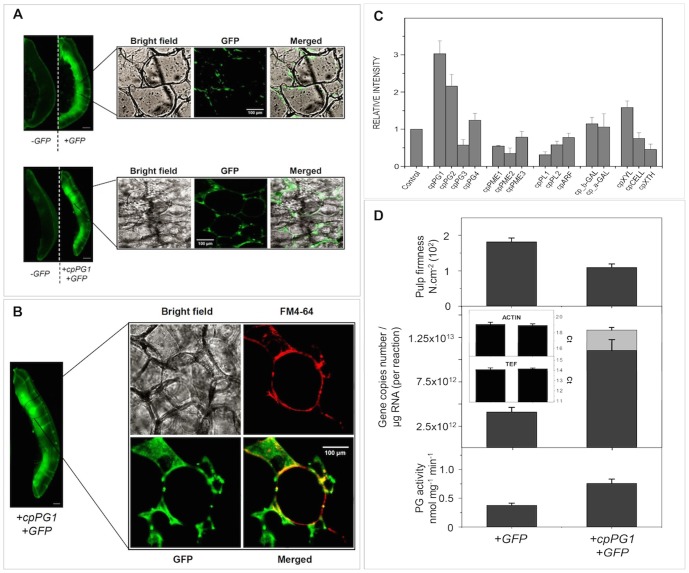
Transient expression of the *cpPG1* gene in the pulp of papaya fruits treated with 1-MCP. Constructs carrying *GFP* or *cpPG1-GFP* genes were agroinfiltrated into papaya fruits treated with 1-MCP, as described in the Materials and Methods. GFP and cpPG1-GFP fusion proteins were transiently expressed and observed with the Versa Doc Gel Imaging System and a confocal laser scanning microscope (**Figures A** and **B**). Contrast with the vital dye FM4-64 shows that the transiently expressed cpPG1-GFP proteins were targeted to the extracellular compartment (**Figure B**). The scale bars in the images of the fruits are 1 cm, and the scale bars in the confocal images indicate the scale. The images are representative of triplicate experiments performed on each agroinfiltrated fruit (*n* = 3). **Figure C** shows the relative mRNA abundance of 15 cell wall-related genes and indicates that *cpPG1* and *cpPG2* may be the main contributors to papaya pulp softening in agroinfiltrated fruits. The expression values for control fruit expressing only GFP (closed vector) were set to 1 for all genes. The absolute quantification of the *cpPG1* mRNA levels in **Figure D** demonstrates that *cpPG1* (gray bar) is transiently expressed. The light gray bar indicates data from transient *cpPG1* expression, the gray bars indicate data from endogenous *cpPG1* expression, and the black bars indicate the threshold cycle values (Ct) for the two genes used as internal controls (*cpACT* and *cp_EF1*).

**Figure 7:**
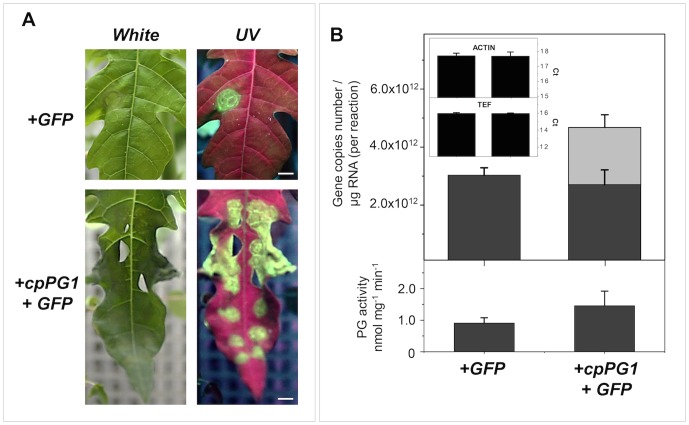
Transient expression of the *cpPG1* gene in papaya leaves. Constructs carrying *GFP* or *cpPG1-GFP* genes were agroinfiltrated into papaya leaves, and the GFP expression in 20-day-old leaves was observed under UV light excitation (**Figure A**). The images are representative of triplicate experiments that were performed on each agroinfiltrated leaf (*n* = 3). The absolute quantification of the *cpPG1* mRNA levels in **Figure B** demonstrate that *cpPG1* (gray bar) is transiently expressed. The light gray bar indicates data from the transient expression of *cpPG1*, the gray bars indicate data from endogenous *cpPG1* expression, and the black bars indicate the threshold cycle values (Ct) for the two genes used as internal controls (*cpACT* and *cp_EF1*). The images are representative of triplicate experiments that were performed on each agroinfiltrated leaf (*n* = 3). The figure bars are scaled to 1 cm.

To verify the role played by this PG gene in pulp softening, 1-MCP-treated fruits were transiently transformed to overexpress *cpPG1*. After three days of agroinfiltration, GFP signals were observed in both the control and *cpPG1-GFP*-transformed fruits, which showed transient cpPG1-GFP protein delivered to the extracellular space (**Figure 6A and B**). The transcript analysis of 15 cell wall-related genes showed that *cpPG1* was up-regulated after treatment, and *cpPG2* also appeared to be affected (**Figure 6C**). The absolute quantitation of the transcript revealed a 20% excess of *cpPG1* mRNA (**Figure 6D**), which was correlated with a two-fold increase in PG activity and pulp softening. Papaya leaves that were agroinfiltrated with the same construct to overexpress *cpPG1* in 1-MCP-treated fruit exhibited a 45% increase in *cpPG1* expression, demonstrated increased activity and survived for only two days, whereas the control leaves survived for six days (**Figure 7**).

## Discussion

Pulp softening is a fast and marked change that occurs in ripening papayas and is denoted by the severe loss of firmness after just a few days. The thinning of the cell wall, which results from the substantial action of enzymes on the polysaccharide structure, causes a loss in pulp tissue rigidity. Considering its complexity, which is derived from various polysaccharide chains and the arrangement of pectic and hemicellulosic fractions, several enzymes are expected to act as a result of the interactions of co-expressed cell wall-related genes.

According to the evaluation of the gene expression profiles of various cell wall enzymes, the depolymerizing capacity is predominately attributed to the increased expression of PGs, β-gal and endoxylanase. This result suggests that the massive solubilization of pectin may be the predominant event, although a minor contribution of the hemicellulose component should not be disregarded, as indicated by the increase in endoxylanase. Notably, the gene encoding PL, an enzyme responsible for pectin depolymerization through a β-elimination reaction, was down-regulated. In fact, WGCNA analysis revealed both positive and negative correlations among the expression patterns of the genes of pectin-acting enzymes, in contrast from what was observed for strawberries, in which a concomitant action of PL and PG might be essential for pulp softening [14], [15]. In addition to PL, the co-expression of PGs was also negatively correlated to that of PMEs. Therefore, the action of papaya PGs does not require the simultaneous removal of pectin methyl groups, which would be consistent with the stable amount of methoxylated pectin during ripening [1], [12], [10], [5].

Another interesting result that emerged from the WGCNA analysis was the co-expression of galactanases, which may act on the heterogalactans of pectins. Alpha-galactosidase genes only marginally contribute to pulp softening [16] and were strongly correlated to PG expression. In contrast, an up-regulated β-galactosidase that is partially responsible for papaya softening [17] demonstrated increased co-expression with genes related to pectin and hemicellulose modifications, such as *GAE6*, *ARF1*, *PL2*, *PL3* and *XTH* [18], [19], [20], [21], [22]. This finding might indicate that *cp_b-GAL* plays a ubiquitous role in papaya plant cell wall mobilization that is not limited to pectin depolymerization during fruit ripening.

The data from the gene expression and co-expression network analysis suggest a cooperative network of cell wall-related genes during papaya pulp softening that is mainly composed of endoPGs, which is consistent with the massive pectin solubilization [5] and the release of uronic acid oligosaccharides [23]. Softening dependence on massive PG action has already been seen in apple fruit, as denoted by the inhibition of softening in transgenic lines with PG suppression [24]. The up-regulation of *cp_EXY1* corroborates previous data reported by Manenoi and Paull [8], and the encoded enzyme accounts for the hydrolysis of β-1,4-D-xylosyl that is bounded to polysaccharides in the cell wall matrix, which are mainly hemicelluloses. However, because cellulase expression decreases during ripening [10], *cp_EXY1* would contribute to the minor disassembly of hemicellulose fractions by the release of heteroglucan chains but not the depolymerization of the cellulose backbone, which is mostly found in a crystalline phase. Therefore, the action of pectinases on less compacted pectin in the amorphous phase would have a more significant effect on cell wall disassembly during ripening.

In addition to a previous observation of the similarities between ripening papayas and *Arabidopsis* hypocotyl gene expression [10], new data from *Arabidopsis* development (young and mature plants – [13] – **Table S1 in File S1**) indicate similar co-expression patterns for several cell wall-related papaya genes. In this regard, the *cpPG1* orthologue from *Arabidopsis* (**AT3G59850**) appears to play a central role in cell wall disassembly because the transgenic expression of this gene and the co-expression of expansin (*EXP2* - AT2G39700 - [25]) markedly changed the pulp firmness and increased the tomato susceptibility to *Botrytis cinerea* [26].

The occurrence of different endoPGs in various fleshy fruit organs has previously been reported [27], [28], [29] and suggests a degree of redundancy or specialization in pectin depolymerization. In this regard, *cpPG1* appears to play a predominant role in papaya softening as it occurs for apple softening [30], [24]. Thus, the more abundant transcript would account for most of the enzymatic activity observed in the fruit pulp, although it was not so well adjusted to the protein peak abundance at the third day after harvest, probably as a result of the interference of other PG proteins, such as cpPG3 and cpPG4, in the assays. Furthermore, since the performed activity assay measures reducing end formation, we cannot exclude a minor contribution of exopolygalacturonases. In relation to *cpPG2*, that gene was separated from *cpPG1* in a distinct clade, and the encoded protein had a *Lys_346_* catalytic residue instead of a *Glu*, which allowed speculation that this product may act differently on pectin depolymerization (**Table S2 and Figure S6 in File S1**).

The transient transformation of papaya demonstrated the effect of *cpPG1* on pulp softening. Treatment with 1-MCP precluded ethylene synthesis and the triggering of PG genes. Fruit agroinfiltrated with pGBPGWG_cpPG1 transiently expressed cpPG1-GFP proteins that were delivered to the extracellular compartment, and the pulp of the fruit softened compared with those that received the empty vector. Notably, the up-regulation of *cpPG2* may reveal some level of responsiveness or dependence on *cpPG1* and insensitivity to ethylene because the gene was expressed during late ripening in non-transformed fruit (**Figure 2**). At the same time, it is possible that other ethylene-independent cell wall-related genes had contributed to partial softening of the pulp. The results of the transient transformation of papaya leaves with pGBPGWG_cpPG1 were similar to those of tobacco leaves that overexpress polygalacturonases [31], which cause cell death and leaf necrosis (**Figure 7A**). Moreover, it is likely that the overexpression of the *cpPG1* gene reduced cell wall adhesion causing disruption of leaf organization as was observed in apple tree leaves constitutively expressing a fruit-specific PG [30]. The above-mentioned results demonstrate that the abundantly expressed *cpPG1* gene may exert profound changes in the papaya cell wall structure and play a central role in pectin disassembly during fruit ripening. The softening of fleshy fruit, particularly pectin-rich fruit, may now be studied through a broader approach by considering a key group of cell wall-related genes and their interactions with each other and other cell wall-related genes, as was demonstrated with papayas.

## Materials and Methods

### Plant material and ripening parameters

Papayas (*Carica papaya* L. cv. Golden) were obtained from a producer in Linhares City, Espírito Santo, Brazil. The fruits were harvested with up to 25% yellow peel (150 days post-anthesis). The fruits were stored in 240 L chambers with controlled temperature and humidity (22°C±0.1°C and 95%, respectively) for seven days. Daily analysis was performed on six fruits (two distinguishable biological replicates from two different seasons). The CO_2_, ethylene and pulp firmness were measured according to Fabi et al. [1], and the remaining pulp was N_2_-frozen, pooled and stored at −80°C. Sections of frozen tissue (12–15 µm) were cut using a refrigerated microtome, stained with toluidine blue (0.05% in 0.1 M phosphate buffer, pH 6.8) and visualized using a light microscope (Zeiss, Germany).

### Bioinformatics and sequence cloning and analysis

The papaya genomic sequences were identified by comparing a partial papaya genome [32] with polygalacturonases from diverse fleshy fruits and *Arabidopsis thaliana* using Blast tools and primers that were designed to flank the coding regions (**Table S3 in File S1**). Papaya pectate lyase (*cpPL1*) and β-galactosidase (*cp_b-GAL*) genes were downloaded from the GenBank database (**DQ660903** and **AF064786**, respectively). For papaya α-L-arabinofuranosidase, a pair of degenerated primers was used to identify part of the coding region (**Table S3 in File S1**). The total RNA was extracted according to Fabi et al. [6], and first-strand cDNAs were synthesized from 1 µg of total RNA using an Improm-II Reverse Transcription System kit (Promega, Madison, WI, USA) and oligo-dT primers. The coding regions were PCR-amplified using KOD Hot Start DNA Polymerase (Merck KGaA, Darmstadt, Germany), and sequences with a high GC content (*cpPG2* and *cpPG3*) were PCR-amplified using KAPA HiFi DNA Polymerase (Kappa Biosystems, Boston, MA, USA). The PCR fragments were digested with *Xho*I and ligated on a pET-45b(+) (Merck KGaA, Darmstadt, Germany) plasmid. After cloning and sequencing, the sequences were analyzed with the TargetP 1.1 tool [33] and the SignalP tool [34]. Finally, the DNA was extracted according to Fabi et al. [6], PCR-amplified as described above, cloned and genomically sequenced.

### Quantitative analysis of gene expression by real time-PCR (qPCR)

Gene expression analyses were performed according to [10] and following the ‘Minimum Information for Publication of Quantitative Real-Time PCR Experiments – MIQE’ [35]. The primer sequences are shown in **Table S4 in File S1**. The gene expression of a previously identified papaya endoxylanase (*cpEXY1* - **AY138968**; [8]) was also analyzed. The actin gene (*ACT*) located on chromosome LG9 contig 1059 (GenBank accession no. ABIM01001059) and the elongation factor 1-alpha gene (*EF1*) located on chromosome LG9 (GenBank accession no. ABIM0101268) were used as internal controls. To evaluate the agroinfiltration experiments using the pGBPGWG vector, a pair of primers enclosing the GFP gene was synthesized (**Table S4 in File S1**). For primer testing and identity confirmation, all of the fragments from the PCR reactions were cloned and sequenced. Real-time PCR was performed using a four-channel Rotor-Gene 3000 multiplexing system (Corbett Research, Sydney, Australia). The non-template controls (NTCs) and melting curve analyses of the amplicons were monitored for all experiments. The threshold cycle (Ct) values (four technical replicates and two biological replicates) were averaged using the Rotor-Gene 3000 software, and the quantification was performed using the relative standard curve method [36]. The results of the standard curves calculations are shown in **Table S5 in File S1**. To assess the gene copy numbers, the corresponding cloned coding regions were used to construct calibration curves (**Table S6 in File S1**). The results are expressed as gene copy numbers/microgram of RNA for each PCR reaction. All of the data were analyzed by one-way ANOVA, and the means were compared using Tukey's test. The statistical analyses were performed using OriginPro version 8 software (OriginLab).

### Comparative biology and co-expression patterns

ClustalW phylogenetic trees [37] were calculated from the alignment of the corresponding proteins with proteins from fleshy fruits and *A. thaliana*. To search for co-expression patterns between genes related to papaya cell wall metabolism, the log_2_ values of the gene expression data from 25 genes were analyzed using the weighted gene co-expression network analysis (WGCNA) in the R package [38]. In summary, pair-wise gene (Pearson) correlations were calculated using gene expression data from genes identified in this study and three previous studies [10], [11], [12]. The correlations and associated *p*-values were calculated using the WGCNA R package and the “corAndPvalue” module. The GENE-E software was loaded with values, and corresponding heat map figures were generated (http://www.broadinstitute.org/cancer/software/GENE-E). A weighted adjacency matrix was then constructed by the WGCNA package using default parameters from the “adjacency” module and a soft-threshold parameter equal to 3, which forces the program to emphasize stronger correlations over weaker ones [39]. The obtained co-expression networks were visualized using Cytoscape [40].

### Heterologous Protein Expression, Purification and Sequencing

Protein expression was performed by transforming Rosetta 2 (DE3) pLysS (Novagen) *E. coli* with a pET-45b(+) construct containing *cpPG*. Bacteria were grown in LB medium (Cm^+^/Amp^+^), and inductions were performed with isopropyl β-D-1-thiogalactopyranoside (IPTG) when the OD_600 nm_ reached 0.6. A control experiment was performed using a closed pET-45b(+) plasmid. Images of polyacrylamide gels were captured using a Versa Doc Gel Imaging System (Bio-Rad, CA, USA). The molecular masses of corresponding bands were estimated by comparing them with standard bands of molecular weight proteins using the Quantity One software (version 4.6.7) (Bio-Rad, CA, USA). The pelleted bacterial suspensions were disrupted using the commercial BugBuster Protein Extraction Reagent (Merck KGaA, Darmstadt, Germany) supplemented with the protease inhibitor cocktail ProteoBlock (Fermentas, Thermo Fisher Scientific Inc., Vilnius, Lithuania). The recombinant proteins were extracted from inclusion bodies using 8 M urea with 24 hours of agitation and purified in a nickel column (Protino NI-TED 1000 - Macherey-Nagel GmbH & Co KG, Düren, Germany) according to the manufacturer's instructions. The samples were analyzed by SDS-PAGE on a 12.5% acrylamide gel [41]. The bands of interest were excised from the gels, and the digestion and protein sequencing were performed according to [42].

### Production of Polyclonal Antibodies

After confirming the identities of the recombinant proteins, 1 mg of the purified protein was separated using SDS-PAGE. The band was excised and the rabbits were immunized according to [43].

### Western Blotting and Enzymatic assays

The total protein from the pulp of papayas was extracted according to Carpentier et al. [44] by adding ProteoBlock (Fermentas) protease inhibitor. The protein concentrations were determined using a 2-D Quant Kit (GE Healthcare, Piscataway, NJ, USA) according to the manufacturer's instructions. Twenty micrograms of total protein were separated by SDS-PAGE and transferred to nitrocellulose membranes (Hybond-ECL, GE Healthcare, Piscataway, NJ, USA). The membranes were incubated with antiserums and secondary anti-rabbit ECL Plex Goat-the-Rabbit Cy5 antibody (GE Healthcare, Piscataway, NJ, USA) according to the manufacturer's instructions. Monoclonal anti-actin for plants (Sigma, #A0480, St. Louis, MO, USA - 1∶5000 dilution) with a secondary anti-mouse ECL Plex Goat-a-Mouse Cy3 antibody (GE Healthcare, Piscataway, NJ, USA) was used as a control experiment. Images of the membranes were captured with a Versa Doc Gel Imaging System (Bio-Rad) using the corresponding channels for Cy5 and Cy3 detection (multiplexing). Images were taken after 120 seconds of exposure for Cy5 and 30 seconds of exposure for Cy3, and the negative image files were obtained with the Quantity One (Bio-Rad) software. The bands observed were compared with the standard molecular weight ECL Plex Fluorescent Rainbow Marker (GE Healthcare). The proteins for enzymatic assays were extracted as previously described [6]. The polygalacturonase activity was assayed according to Fabi et al. [6].

### Transient gene expression of *cpPG1* in papaya fruits and leaves

The *cpPG1* gene was transiently expressed in papaya fruits and leaves by agroinfiltration. The binary vector used in the experiments was pGBPGWG expressing green fluorescent protein (GFP) under the regulation of the 35S promoter (GenBank accession number **AM884372**), and the *cpPG1* ORF was cloned into the vector by recombination, as previously described by Zhong et al. [45]. The agroinfiltration of the papaya fruits was performed according to Spolaori et al. [46]. *Agrobacterium tumefaciens* GV3101 containing a pSoup plasmid (Tet^+^/Gent^+^) was transformed with a binary vector that enclosed *cpPG1* ORF (pGBPGWG_cpPG1 – Cm^+^/Kan^+^) and used in both experiments. Moreover, bacterial suspensions were co-infiltrated (1∶1) with *A. tumefaciens* GV2260 that was transformed with the 35S: p19 binary vector (Rif^+^/Kan^+^) to decrease the post-transcriptional gene silencing of exogenous *cpPG1* [47].

One day after harvesting, the papaya fruits were treated with 100 ppb of 1-methylcyclopropene (1-MCP) for 12 hours to reduce *cpPG1* endogenous gene expression, as previously described [1]. After treatment, three fruits (*n* = 3) were agroinfiltrated using sterile syringes and a short needle (1 mL bacteria suspension per 4 cm^2^). The fruits were allowed to ripen at 22°C for a 16-h photoperiod. A previous test using a mixture of *Agrobacterium* and methylene blue was conducted to calculate the extent of agroinfiltration. Three days post-infiltration, the fruits were sliced and photographed using a Versa Doc Gel Imaging System (Bio-Rad) with parameters for GFP detection. The pulp firmness was measured according to Fabi et al. [1]. The remaining slices were N_2_-frozen for future gene expression quantification. A control experiment (*n* = 3) was similarly conducted using the pGBPGWG-closed vector. Sections of pulp were manually cut, stained with FM4-64 vital dye and analyzed using a confocal laser-scanning microscope (Zeiss, Jena, Thuringia, Germany, LSM 510). The images were processed using the LSM image browser software.

The agroinfiltration of young papaya leaves (20 days and 50 days after germination) was performed according to Boudart et al. [31]. The abaxial spaces of the leaves (*n* = 6) were agroinfiltrated using sterile syringes (without the needle). The plants were maintained at 22°C for a 16 h photoperiod, and the leaves were collected 2 days post-infiltration (20-day-old leaves). A control experiment (six leaves) was similarly conducted using the pGBPGWG closed vector.

## Supporting Information

File S1 **These are the legends for Supporting Tables / Figures presented in File S1.** Table S1. Cell wall-related genes from ripe papaya and mature *A. thaliana* plant. Table S2. Similarity percentage of amino acid from papaya and other plants PGs. Table S3. Nucleotide sequences used in PCR reactions. Table S4. Nucleotide sequences used in qPCR. Table S5. Calibration curves for relative gene expression. Table S6. Calibration curves for absolute gene expression. Figure S1. Up-regulation of cell wall-related genes during papaya ripening. Real-time PCR (qPCR) was used to determine the absolute quantitation of the mRNA levels of various genes during papaya ripening. The quantification is represented by the column height. The error bars on each column indicate the SD from four technical replicates from samplings I and II. The different letters represent samples that were significantly different from those collected on other days post-harvest (within the same gene) as determined by one-way ANOVA and Tukey's test (α<0.05, *n* = 4). **Figure B** shows the threshold cycle values (Ct) for the two genes used as internal controls (actin gene – *cpACT* and elongation factor 1-alpha gene - *cp_EF1*). Figure S2. Genomic and mRNA organization of different PGs from papaya fruit. Grey boxes represent coding regions (exons), while black lines represent non-coding regions (introns). White boxes represent the mRNA sequences concatenated from the above compared exons. Figure S3. Unrooted phylogram encompassing PGs from papaya, *Arabidopsis* and several other plant organisms. A phylogenetic tree was calculated using the neighbor-joining method based on the ClustalW alignment of the deduced amino acid sequences. The putative signal peptide from all of the proteins was removed from the sequence. The following proteins and their corresponding GenBank IDs were used: *A. thaliana 1, 2 and 3* (**NP_191544**, **NP_191310**, **NP_187454**), *P. persica 1 and 2* (**AAC64184**, **CAA54448**), *P. communis 1* and *2* (**CAH18935**, **BAC22688**), *D. carota* (**BAC87792**), *S. lycopersicum 1 and 2* (**ABW38780**; **AC28947**), *C. melo 1* and *2* (**AAC26510**; **AAC26512**), *V. vinifera 1* and *2* (**XP_002263164**, **XP_002282759**), *B. napus* (**CAA65072**), *G. Max 1* and *2* (**ABC70314**, **AAL30418**), *D. kaki* (**ACJ06506**), *R. communis 1*, *2* and *3* (**XP_002529090**, **XP_002529616**, **XP_002517823**), *A. deliciosa* (**P35336**), *O. europaea* (**ACA49228**), *P. trichocarpa 1*, *2* and *3* (**XP_002331621**, **XP_002322711**, **XP_002313308**) and *C. papaya 1, 2, 3* and *4* (**FJ007644**, **GQ479791**, **GQ479794** e **GQ479795**). The values for the branch lengths are based on the scale bar; there are 0.1 residue substitutions per site. Letters **A** and **B** show the two distinct clades resulting from the phylogenetic analyses. Figure S4. Recombinant proteins sequencing. Figures show the query sequences of cpPG1 (**A**) protein, and the correspondent peptides that were visualized in protein sequencing. Residues highlighted by gray boxes and black letters are from expression vector; residues highlighted by black boxes and white letters are those obtained in protein sequencing. The histidine tags are underlined and asterisks indicate the end of protein. Figure S5. Expression of recombinant proteins from papaya pulp. The figures show representative gels from heterologous expression of *cpPG1* (**A**) gene. **Figure A.** Proteins profiles from bacteria carrying pET45-*cpPG1* before IPTG induction (PG), after 16 hs growth non-induced (PG-NI) and after 16 hs growth IPTG-induced (PG-I); Ni-purified recombinant proteins can be seen after properly elution (E 1 and 2). Proteins markers are highlighted with weight numbers. **Figure B.** The figure displays the hybridization of antiserum for cpPG1 recombinant protein. The arrow indicates the molecular weight for commercially available protein markers. The quantities of proteins are also indicated. Figure S6. Protein alignment of four distinct papaya PGs. The corresponding proteins from *cpPG1* (PG1 - **FJ007644**), *cpPG2* (PG2 - **GQ479791**), *cpPG3* (PG3 - **GQ479794**) and *cpPG4* (PG4 - **GQ479795**) genes were aligned using ClustalW and viewed with BOXSHADE program and then manually edited. Gaps were introduced to optimize alignment. Identic amino acids are highlighted by black boxes and white letters, similar amino acids are highlighted by grey boxes and white letters and different amino acids are highlighted by white boxes and black letters. The conserved domain of glycosil hydrolases from family 28 (**PS00502**) is indicated by a dotted-line box. The probable catalytic residues are indicated by asterisks, being possible to observe the cpPG2 protein has a *Glu_346_* instead of a *Lys_346_* as the other three polygalacturonases have.
(ZIP)
