# Supplementary material for: Analysis of Papaya Cell Wall-Related Genes during Fruit Ripening Indicates a Central Role of Polygalacturonases during Pulp Softening
Source: PLoS One. 2014 Aug 27;9(8):e105685. doi: 10.1371/journal.pone.0105685 (PMC4146514; doi:10.1371/journal.pone.0105685)
Supplement: File S1 — These are the legends for Supporting Tables / Figures presented in File S1. Table S1. Cell wall-related genes from ripe papaya and mature A. thaliana plant. Table S2. Similarity percentage of amino acid from papaya and other plants PGs. Table S3. Nucleotide sequences used in PCR reactions. Table S4. Nucleotide sequences used in qPCR. Table S5. Calibration curves for relative gene expression. Table S6. Calibration curves for absolute gene expression. Figure S1. Up-regulation of cell wall-related genes during papaya ripening. Real-time PCR (qPCR) was used to determine the absolute quantitation of the mRNA levels of various genes during papaya ripening. The quantification is represented by the column height. The error bars on each column indicate the SD from four technical replicates from samplings I and II. The different letters represent samples that were significantly different from those collected on other days post-harvest (within the same gene) as determined by one-way ANOVA and Tukey's test (α<0.05, n = 4). Figure B shows the threshold cycle values (Ct) for the two genes used as internal controls (actin gene – cpACT and elongation factor 1-alpha gene - cp_EF1). Figure S2. Genomic and mRNA organization of different PGs from papaya fruit. Grey boxes represent coding regions (exons), while black lines represent non-coding regions (introns). White boxes represent the mRNA sequences concatenated from the above compared exons. Figure S3. Unrooted phylogram encompassing PGs from papaya, Arabidopsis and several other plant organisms. A phylogenetic tree was calculated using the neighbor-joining method based on the ClustalW alignment of the deduced amino acid sequences. The putative signal peptide from all of the proteins was removed from the sequence. The following proteins and their corresponding GenBank IDs were used: A. thaliana 1, 2 and 3 (NP_191544, NP_191310, NP_187454), P. persica 1 and 2 (AAC64184, CAA54448), P. communis 1 and 2 (CAH18935, BAC22688), D. carota ( [file pone.0105685.s001.zip › Table S3 - primers regular PCR.docx]

| **Table S3.** Nucleotide sequences used in PCR reactions. | | |
| --- | --- | --- |
| *Gene/Specificity* | *Primer name* | *Sequence (5’→3’)* |
| OligodT | >oligodT | (T)_18_(A/C/G/T)(A/C/G/T) |
| T7_promoter | >T7 | TAA TAC GAC TCA CTA TAG GG |
| *cpPG1* | >PG1_f | ATG ACG ACA ATC CGC TCT CAC AAC |
|  | >PG1-SP_f | GTC AAC CCA CAA CGT CGT GGC C |
|  | >PG1_XhoI_r | GAG A**CT CGA G**TC ACA AGC AAC TAT TGG GC |
| *cpPG2* | >PG2_f | ATG GCT GTT CTA TAT TAT GAT |
|  | >PG2-SP_f | GTT CCA TGG CGA GCG TCG TC |
|  | >PG2_XhoI_r | GAG A**CT CGA G**CT AAA GCT CGT CAT GTT TA |
| *cpPG3* | >PG3_f | ATG CCG ATG GGA AAA GAG CAT |
|  | >PG3-SP_f | GTA CCA GGA CGA GCA ATC TCC |
|  | >PG3_XhoI_r | GAG A**CT CGA G**TC AAT TAT TAA AGC AAT AC |
| *cpPG4* | >PG4_f | ATG GCA AAC TTA ATG GTA AAC TCA G |
|  | >PG4-SP_f | TAA GAC ATC ATC AGC AAG CCC T |
|  | >PG4_XhoI_r | GAG A**CT CGA G**TC ATT GGT CAA TAT TTG CA |
| cp_b-GAL | >GAL_f | GGC ACG AGA AAC ACA CTC AAC |
|  | >GAL-SP_f | GGT GTC TTA CGA CCA TAA AGC |
|  | >GAL_XhoI_r | GAG A**CT CGA G**CT ACA CAA CTC TTT TCA CC |
| cpPL | >PL_f | GGA GGA GCA TAC GTA ATA GTA |
|  | >PL-SP_f | GCG TAA TAG TAC GGA AAG GCG |
|  | >PL_XhoI_r | GAG A**CT CGA G**TC AAC ATT GGG AAC CAT AG |
| cpARF | >ARF_f | ATG CTT TGG GCA GAT TAT GCG |
|  | >ARF-SP_f | GGA AGT TGG AGC GAA CGT TAC |
|  | >ARF_XhoI_r | GAG A**CT CGA G**TC AAG TGG ACG ATT TTG AG |
| ** XhoI* restriction site are in bolded and underlined letters. | | |
