# Supplementary material for: Analysis of Papaya Cell Wall-Related Genes during Fruit Ripening Indicates a Central Role of Polygalacturonases during Pulp Softening
Source: PLoS One. 2014 Aug 27;9(8):e105685. doi: 10.1371/journal.pone.0105685 (PMC4146514; doi:10.1371/journal.pone.0105685)
Supplement: File S1 — These are the legends for Supporting Tables / Figures presented in File S1. Table S1. Cell wall-related genes from ripe papaya and mature A. thaliana plant. Table S2. Similarity percentage of amino acid from papaya and other plants PGs. Table S3. Nucleotide sequences used in PCR reactions. Table S4. Nucleotide sequences used in qPCR. Table S5. Calibration curves for relative gene expression. Table S6. Calibration curves for absolute gene expression. Figure S1. Up-regulation of cell wall-related genes during papaya ripening. Real-time PCR (qPCR) was used to determine the absolute quantitation of the mRNA levels of various genes during papaya ripening. The quantification is represented by the column height. The error bars on each column indicate the SD from four technical replicates from samplings I and II. The different letters represent samples that were significantly different from those collected on other days post-harvest (within the same gene) as determined by one-way ANOVA and Tukey's test (α<0.05, n = 4). Figure B shows the threshold cycle values (Ct) for the two genes used as internal controls (actin gene – cpACT and elongation factor 1-alpha gene - cp_EF1). Figure S2. Genomic and mRNA organization of different PGs from papaya fruit. Grey boxes represent coding regions (exons), while black lines represent non-coding regions (introns). White boxes represent the mRNA sequences concatenated from the above compared exons. Figure S3. Unrooted phylogram encompassing PGs from papaya, Arabidopsis and several other plant organisms. A phylogenetic tree was calculated using the neighbor-joining method based on the ClustalW alignment of the deduced amino acid sequences. The putative signal peptide from all of the proteins was removed from the sequence. The following proteins and their corresponding GenBank IDs were used: A. thaliana 1, 2 and 3 (NP_191544, NP_191310, NP_187454), P. persica 1 and 2 (AAC64184, CAA54448), P. communis 1 and 2 (CAH18935, BAC22688), D. carota ( [file pone.0105685.s001.zip › Table S4 - primers qPCR.docx]

| **Table S4.** Nucleotide sequences used in qPCR. | | |
| --- | --- | --- |
| *Gene* | *Primer name* | *Sequence (5’→3’)* |
| *cpPG1* | >pg1_f | TGG TGG TGC GTA TAG ATG GA |
|  | >pg1_r | ACA AAA CCC AGT ACC CAC CA |
| *cpPG2* | >pg2_f | TCC TGA AGC TCA CCC TTC AT |
|  | >pg2_r | CCT CAA TGC CTT TGA AGC TC |
| *cpPG3* | >pg3_f | TTG GAG GGC AGC TTG TTT AG |
|  | >pg3_r | CAC CCA AGC CTT TAT TGT TCC |
| *cpPG4* | >pg4_f | ATG GCC TTA CAG ACT CCA CA |
|  | >pg4_r | TGG TGG CAG GGT TTA TTG AG |
| *cp_b-GAL* | >gal_f | GTG CTT GCA ACT ATG CTG GA |
|  | >gal_r | ATA GGT TCG CAG TTG GGT TG |
| *cpPL* | >pl_f | TTC CCT GTG GGC TTA CAA TC |
|  | >pl_r | CAT GTT CTT GTC CTG CGT GT |
| *cpARF* | >arf_f | AGG TGG CTG TTT TGT TGA GG |
|  | >arf_r | TCT CTT CCC AAG GTC CAA TG3 |
| *cpXYL* | >xyl_f | GCT TCC GCT GTG TTT TAT GG |
|  | >xyl_r | ATG ATT GGA TCG ACC TCA GC |
| *cpACT* | >act_f | CGT GAC CTT ACT GAT CAC TTG |
|  | >act_r | GTC AAG GGC AAT GTA AGA CAG |
| *cpEF1* | >ef1_f | GTT AAG AAC GTT GCC GTG AAG |
|  | >ef1_r | ATG TGA AGT TGG CTG CTT CCT |
| *GFP* | >gfp_f | GAA CGG CAT CAA GGT GAA C |
|  | >gfp_r | TGC TCA GGT AGT GGT TGT C |
