# Supplementary material for: Analysis of Papaya Cell Wall-Related Genes during Fruit Ripening Indicates a Central Role of Polygalacturonases during Pulp Softening
Source: PLoS One. 2014 Aug 27;9(8):e105685. doi: 10.1371/journal.pone.0105685 (PMC4146514; doi:10.1371/journal.pone.0105685)
Supplement: File S1 — These are the legends for Supporting Tables / Figures presented in File S1. Table S1. Cell wall-related genes from ripe papaya and mature A. thaliana plant. Table S2. Similarity percentage of amino acid from papaya and other plants PGs. Table S3. Nucleotide sequences used in PCR reactions. Table S4. Nucleotide sequences used in qPCR. Table S5. Calibration curves for relative gene expression. Table S6. Calibration curves for absolute gene expression. Figure S1. Up-regulation of cell wall-related genes during papaya ripening. Real-time PCR (qPCR) was used to determine the absolute quantitation of the mRNA levels of various genes during papaya ripening. The quantification is represented by the column height. The error bars on each column indicate the SD from four technical replicates from samplings I and II. The different letters represent samples that were significantly different from those collected on other days post-harvest (within the same gene) as determined by one-way ANOVA and Tukey's test (α<0.05, n = 4). Figure B shows the threshold cycle values (Ct) for the two genes used as internal controls (actin gene – cpACT and elongation factor 1-alpha gene - cp_EF1). Figure S2. Genomic and mRNA organization of different PGs from papaya fruit. Grey boxes represent coding regions (exons), while black lines represent non-coding regions (introns). White boxes represent the mRNA sequences concatenated from the above compared exons. Figure S3. Unrooted phylogram encompassing PGs from papaya, Arabidopsis and several other plant organisms. A phylogenetic tree was calculated using the neighbor-joining method based on the ClustalW alignment of the deduced amino acid sequences. The putative signal peptide from all of the proteins was removed from the sequence. The following proteins and their corresponding GenBank IDs were used: A. thaliana 1, 2 and 3 (NP_191544, NP_191310, NP_187454), P. persica 1 and 2 (AAC64184, CAA54448), P. communis 1 and 2 (CAH18935, BAC22688), D. carota ( [file pone.0105685.s001.zip › Table S6 - calibration curve absolute.docx]

| **Table S6.** Calibration curves for absolute gene expression. | | |
| --- | --- | --- |
| *Name of the gene* | *y=ax+b* | *R^2^* |
| *cpPG1* | -2.135x - 0.2096 | 0.9980 |
| *cpPG2* | -2.720x - 1.0957 | 0.9957 |
| *cpPG3* | -2.775x - 1.3010 | 0.9933 |
| *cpPG4* | -2.610x - 0.7186 | 0.9984 |
| *cp_b-GAL* | -3.215x - 1.6077 | 0.9998 |
| *cpXYL* | -2.375x - 0.2218 | 0.9998 |
| *GFP* | -2.610x - 1.8914 | 0.9984 |
